# *C9orf72* Hexanucleotide Repeat in Huntington-Like Patients: Systematic Review and Meta-Analysis

**DOI:** 10.3389/fgene.2020.551780

**Published:** 2020-11-02

**Authors:** Carlos Alva-Diaz, Christoper A. Alarcon-Ruiz, Kevin Pacheco-Barrios, Nicanor Mori, Josmel Pacheco-Mendoza, Bryan J. Traynor, Andrea Rivera-Valdivia, Pongtawat Lertwilaiwittaya, Thomas D. Bird, Mario Cornejo-Olivas

**Affiliations:** ^1^Facultad de Ciencias de la Salud, Universidad Científica del Sur, Lima, Peru; ^2^Unidad de Investigación Para la Generación y Síntesis de Evidencias en Salud, Vicerrectorado de Investigación, Universidad San Ignacio de Loyola, Lima, Peru; ^3^Servicio de Neurología, Departamento de Medicina, Hospital Nacional Daniel Alcides Carrión, Callao, Peru; ^4^Unidad de Investigación en Bibliometria, Vicerrectorado de Investigación, Universidad San Ignacio de Loyola, Lima, Peru; ^5^Neuromuscular Diseases Research Section, Laboratory of Neurogenetics, National Institute of Aging, NIH, Bethesda, MD, United States; ^6^Neurogenetics Research Center, Instituto Nacional de Ciencias Neurológicas, Lima, Peru; ^7^Fogarty Northern Pacific Global Health Fellows Program, Seattle, WA, United States; ^8^Fogarty Interdisciplinary Cerebrovascular Diseases Training Program in South America, Lima, Peru; ^9^Department of Medicine, Faculty of Medicine Siriraj Hospital, Mahidol University, Bangkok, Thailand; ^10^Department of Neurology, University of Washington, Seattle, WA, United States; ^11^Geriatric Research, Education, and Clinical Center, VA Puget Sound Health Care System, Seattle, WA, United States; ^12^Center for Global Health, Universidad Peruana Cayetano Heredia, Lima, Peru

**Keywords:** C9orf72, Huntington like disorders, systematic review, chorea, prevalence studies

## Abstract

**Introduction:** Patients with Huntington-Like disorders (HLD) comprise a variety of allelic disorders sharing a Huntington phenotype. The hexanucleotide repeat expansion of the *C9orf72* gene could explain part of the HLD etiology. We aimed to conduct a systematic review and meta-analysis looking for the frequency of the hexanucleotide repeat expansion of the *C9orf72* gene in HLD patients.

**Methods:** The protocol was registered on the International Prospective Register of Systematic Reviews database (PROSPERO) (registration number: CRD42018105465). The search was carried out in Medline, Scopus, Web of Science, and Embase in April 2018, and updated in July 2020. Observational studies reporting patients with HLD carrying the hexanucleotide repeat expansion in the *C9orf72* gene were selected and reviewed; this process was duplicated. The cutoff threshold for considering the hexanucleotide expansion as a pathogenic variant was equal to or >30 G_4_C_2_ repeats. Cases with intermediate alleles with 20–29 repeat are also analyzed. Pooled frequency and 95% CI were calculated using random-effects models.

**Results:** Nine out of 219 studies were selected, reporting 1,123 affected individuals with HLD. Among them, 18 individuals carried *C9orf72* expansion, representing 1% (95% CI: 0–2%, *I*^2^ = 0%) of the pooled frequency. Seven selected studies came from European centers, one was reported at a US center, and one came from a South-African center. We identified five individuals carrying intermediate alleles representing 3% (95% CI: 0–14%, *I*^2^ = 78.5%).

**Conclusions:** The frequency of *C9orf72* unstable hexanucleotide repeat expansion in HLD patients is very low. Further studies with more accurate clinical data and from different ethnic backgrounds are needed to confirm this observation.

## Introduction

Huntington disease is an autosomal dominant neurodegenerative disorder caused by an abnormal CAG repeat expansion within the *HTT* gene (4p16.3) (Schneider and Bird, 2016). The estimated global prevalence of Huntington disease is 5.5 per 100,000 inhabitants (Baig et al., 2016; Rawlins et al., 2016), and the disease is clinically characterized by movement disorders (mainly chorea), cognitive decline, and behavioral disturbances, with typical age at onset in young adulthood (McColgan and Tabrizi, 2017). The CAG-*HTT* mutation is not present in ~1% of Huntington disease cases, and this critical subgroup of cases are known as Huntington-like disorders (HLD) (Wild and Tabrizi, 2007). The genetic causes of the HLD phenotype include the genes involved in spinocerebellar ataxia (SCA) type 17, familial prion disease, Friedreich ataxia, dentatorubral-pallidoluysian atrophy, and *C9orf72* related disorders (Schneider and Bird, 2016). Nevertheless, most people with HLD remain undiagnosed.

The *C9orf72* gene codifies for a protein that regulates endosomal trafficking, autophagy, and endocytic transport (Farg et al., 2014). A large, unstable hexanucleotide repeat expansion in this gene accounts for ~10% of amyotrophic lateral sclerosis cases and a similar percentage of frontotemporal dementia cases (DeJesus-Hernandez et al., 2011; Mori et al., 2013). Interestingly, the *C9orf72* pathogenic expansion has also been nominated as a cause of HLD, with some authors reporting a frequency of 5% among European ancestry populations (Hensman Moss et al., 2014a; Kostic et al., 2014; Koutsis et al., 2015; Martins et al., 2018a). In contrast, the *C9orf72* repeat expansion has not been found as a cause of HLD in Latin America and the Caribbean (Walker et al., 2018), providing *prima facie* evidence of differing etiologies of HLD across global populations.

Previous systematic reviews found a 1.6 and 2% prevalence of *C9orf72* repeat expansion in HLD patients (Marogianni et al., 2019; Rikos et al., 2020). However, we strictly followed PRISMA guidelines for systematic reviews and analyzed cases with intermediate alleles (<30 repeat range), which are cataloged as unclear clinical significance (Fedotova et al., 2016; Ida et al., 2018). To explore this further, we performed a systematic review of *C9orf72* hexanucleotide repeat expansion, including fully penetrant and intermediate alleles, associated with HLD reported in the literature.

## Methods

### Literature Search and Study Selection

A systematic review of the literature was conducted following PRISMA guidelines (Liberati et al., 2009; Moher et al., 2009), and the study protocol was registered in PROSPERO (CRD42018105465). We searched in Medline, Scopus, Web of Science, and Embase databases until 25th April 2018 using a research strategy with the search terms: “Huntington Disease-Like,” “Huntington Disease Phenocopy,” “Huntington-Like,” “Huntington Disease,” “Huntington Chorea,” and “C9orf72 protein.” The full research strategy is shown in Supplementary Material 1. Following the initial search, new relevant studies were found on social media, prompting an updated search to be conducted on July 15th, 2020 (Pubmed). Additionally, we reviewed bibliographic references of the included studies in the present review.

Selected studies for this review included those reporting individuals with a HLD diagnosis that screened negative for pathogenic *HTT* CAG repeat expansions and included the *C9orf72* hexanucleotide repeat expansion frequency. We used a cutoff of 30 or more repeats plus the typical sawtooth pattern to identify a pathogenic repeat expansion (Renton et al., 2011; Ida et al., 2018). Additionally, we analyzed some studies reporting intermediate alleles (20–29 repeat range) of unclear clinical significance (Fedotova et al., 2016; Ida et al., 2018). We did not exclude studies by design, date, or language.

Two independent researchers selected the studies. Duplicates were eliminated before selection. The remaining citations were reviewed independently by two researchers (CAR and CAD) in terms of titles and abstracts. Discrepancies between reviewers were resolved by a third reviewer (KPB). After the initial review, the two primary reviewers independently assessed the studies's full text, and the third reviewer resolved the discrepancies.

### Quality Evaluation

The selected studies' methodological quality was evaluated utilizing the tool Newcastle Ottawa quality assessment scale for case-control studies (Stang, 2010), and the adapted version for cross-sectional studies (Modesti et al., 2016). This scale evaluates an observational study according to three categories: (a) Selection (four items, with a maximum of four stars in case-control studies and five stars in cross-sectional studies). For cross-sectional studies, this category assesses sample representativeness to the target population (or complete inclusion of population), justification of satisfactory sample size, relevant selection criteria (exclusion of other relevant diseases), and an adequate ascertainment of exposure (*C9orf72* hexanucleotide repeat expansion). For case-control studies, this category assesses for correct case definition (HLD diagnosis), sample representativeness of the case group to the target population (or complete inclusion of population), selection of community-based control, and an adequate control definition. (b) Comparability (one item, with a maximum of two stars). This item assesses the analysis which controls for confounding factors in both study designs. (c) The outcome for cross-sectional studies (maximum of three stars), which assesses the correct outcome definition (HLD diagnosis) and the correct explanation of the statistical test. (c) Exposure for case-control studies (maximum of three stars), which assesses for an adequate ascertainment of exposure (*C9orf72* hexanucleotide repeat expansion), confirming that the same methods were used for the ascertainment of exposure in case and control groups, and the equal exposure assessment in both control and cases groups. In total, each cross-sectional study could get 10 stars, and each case-control study could get nine stars. A high-quality study was considered as one with six or more stars for cross-sectional studies and five or more stars in case-control studies, as a sum of the three categories. Low-quality studies obtained a lower count of stars than the threshold in both cases.

### Data Extraction

Data extraction from each selected study was conducted independently by two reviewers (CAR and CAD) and then revised by a third reviewer (KPB). A data extraction card was used for each selected study, obtaining information on the frequency of the *C9orf72* hexanucleotide repeat expansion in patients with HLD. We also extracted data referring to the characteristics of the study and the study population: author, year of publication, funding of the study, number of patients evaluated, the proportion of patients with a genetic variant rate with equal or more than 30 repeats, and with 20–29 repeats, clinical characteristics, gender, and the average age at onset. The extracted data were tabulated, coded, and then imported into a datasheet for analysis.

### Statistical Analyses

Each study's results were expressed as a proportion with corresponding 95% confidence intervals, as recommended for small sample sizes studies (Nyaga et al., 2014). These data were synthesized using meta-analysis with random effects model since there might be more than one true effect of the *C9orf72* hexanucleotide repeat expansion for the HLD patients, as per recommendations for prevalence meta-analysis (Aromataris and Munn, 2020). Additionally, we used a double arcsine transformation to calculate the pooled prevalence due to confidence interval limits close to 0% (Barendregt et al., 2013). Heterogeneity was assessed using an I^2^ statistic, and we considered an I^2^ < 40% to be low, 30–60% to be moderate, 50–90% to be substantial, and 75–100% as considerable (Deeks et al., 2019). Additionally, we conducted a sensitivity analysis by removing studies with *C9orf72* hexanucleotide repeat expansion frequency of 0% and performed subgroup analysis by grouping the studies according to design. The data were processed using the “*metaprop*” command (Nyaga et al., 2014) in Stata version 16.

Publication bias was not statistically assessed as the number of studies pooled for each meta-analysis was <10 (Deeks et al., 2019).

### Ethics Review Approval

This study was approved by the Ethics Committee at *Instituto Nacional de Ciencias Neurológicas*, Lima, Peru. No personal information was used in this analysis, and no attempt was made to identify subjects. All data used in this study were anonymous.

## Results

### Studies Characteristics

**Figure 1:**
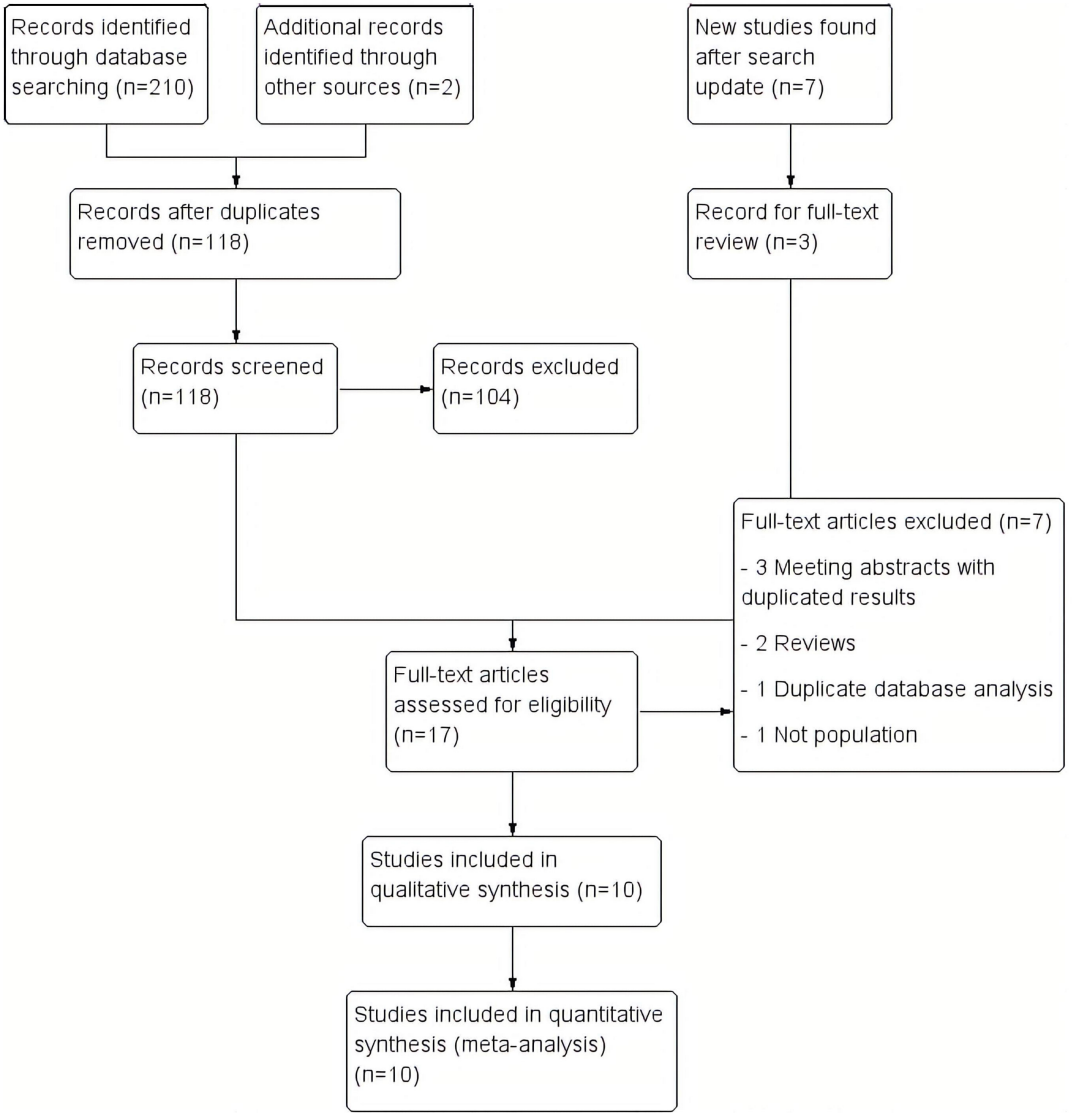
Study flow diagram. We included 210 records in the initial review, 7 more in the updated review and 2 more from other sources. Finally, we included 10 studies in the synthesis.

A total of 210 studies were identified during the initial search. Ninety-four duplicated studies were removed, and 118 studies were reviewed by title and abstract. Of these, 104 studies were excluded due to incorrect type of study (clinical case without population frequency, non-original letter to the editor, and review studies), and incorrect subjects (including population and frequency). Twelve studies were identified for full-text review. The updated search conducted ~2 years later yielded seven additional titles from Pubmed. Three of these new findings were included in the full-text review, and finally, all of them were included in the systematic synthesis (Baine et al., 2018; Ida et al., 2018). Two additional studies were identified through other sources. Then, after the full-text review, seven studies were excluded (Beck et al., 2013; Hensman Moss et al., 2014b; Abramycheva et al., 2015; Dolzhenko et al., 2017; Kartanou et al., 2017; Martins et al., 2018b; Marogianni et al., 2019) (Supplementary Table 1). Finally, 10 studies were included in the meta-analysis (Hensman Moss et al., 2014a; Kostic et al., 2014; Koutsis et al., 2015; Fedotova et al., 2016; Mariani et al., 2016; Baine et al., 2018; Ida et al., 2018; Martins et al., 2018a,b; Kaur et al., 2020; Rikos et al., 2020). A flow diagram of the analysis is presented in Figure 1. No additional studies were retrieved from the citations.

Descriptive information on each article included in the meta-analysis is provided in Table 1. Cumulatively, the 10 studies reported data on the presence or absence of the *C9orf72* repeat expansion in 1,123 patients with HLD. Only three studies, reporting on 287 patients, provided information on the *C9orf72* intermediate allele (defined as 20–29 repeats). The publication years of the included studies ranged from 2014 to 2020.

**Table 1:** Descriptive information on each article included in the systematic review.

**References**	**Country**	***n***	**Population**	**(G_**4**_C_**2**_)n *C9orf72* ratio (≥30 repeats)**	**(G_**4**_C_**2**_)n *C9orf72* ratio (20–29)**	Clinical characteristics and family history^*^	Gender^*^	Median age at onset^*^	**(G_**4**_C_**2**_)n *C9orf72* diagnosis method**
Hensman Moss et al. (2014a)	United Kingdom	514	HLD patients who underwent negative diagnostic genetic testing for HD at NHNN	1.95% (*n =* 10)	–	Early psychiatric, and behavioral problems. Chorea, dystonia, myoclonus, tremor, rigidity, and bradykinesia. Memory problems and cognitive impairment 7 out 10 patients had positive family history	NA	42.7 years	Fragment length analysis on an 3730xl automated sequencer. Then, analysis of repeat primed PCR electropherograms using Peak Scanner. Expansions were identified and put forward for Southern blotting with subsequent hybridization
Kostic et al. (2014)	Serbia	39	HLD patients who underwent negative diagnostic genetic testing for HD and assessed by a specialist. All patients have Serbian ethnic background	2.56% (*n =* 1) 270 repeats	-	Chorea, oro-buccolingual dyskinesia and motor impersistence. Memory problems and change in personality Mother had unspecified dementia	Female	51 years	Fragment length analysis on an 3500xl genetic analyzer. Then, analysis of repeat primed PCR. Expansions were identified and put forward for Southern with subsequent hybridization
Koutsis et al. (2015)	Greece	40	HLD patients who underwent negative diagnostic genetic testing for HD at Neurogenetics Unit at the 1st Department of Neurology, University of Athens Medical School	5% (*n =* 2) >30 repeats	-	Change in personality, chorea, and parkinsonism. Problems with concentration and memory. Spastic dysarthria and emotional lability One patient with her father had amyotrophic lateral sclerosis, her uncle had parkinsonism, and her aunt and grandfather had dementia. Other patient with no family history	Female and male	42.5 years	“Sizing polymerase chain reaction (PCR)” and repeat primed PCR using KAPA Long-Range DNA polymerase. Allele identification and scoring were carried out using GeneScan v3.7 software
Fedotova et al. (2016)	Russia	31	Russian cohort of HLD patients	0%	9.68% (*n =* 3)	NA	NA	NA	Genomic DNA isolated with Wizar genomic DNA purification kit analysis of repeat primed PCR. Allele identification with sequencing analysis v.5.2 software.
Mariani et al. (2016)	France	23	HLD patients who underwent negative diagnostic genetic testing for HD at Department of Genetics of the Pitié-Salpêtrière University Hospital (Paris, France)	0%	–	NA	NA	NA	Repeat expansions in *C9orf72* were detected by repeat-primed PCR. The PCR products were resolved on a sequencer and analyzed with a software program (GeneMapper)
Martins et al. (2018a)	Portugal	20	HLD patients cohort from a tertiary center	5% (*n =* 1) 140 repeats	5% (*n =* 1)	Psychotic episodes, and behavioral disfunction. Hand tremor, symmetrical parkinsonism, dystonia, and orolingual chorea. Progressive cognitive decline Older sister and daughter had severe psychiatric disease	Female	50 years	First, repeat-primed PCR amplification and then, fragment length analysis on an ABIPrism 3130xl sequencer and fragments were analyzed through the GeneMapper software
Baine et al. (2018)	South Africa	97	Database of black South African patients' samples	0%	–	NA	NA	NA	Repeat expansions in *C9orf72* were amplified by repeat-primed PCR. Then, PCR products were analyzed by capillary electrophoresis on the 3130xl genetic analyzer, and repeat sizes estimated using GeneMapper software
Ida et al. (2018)	North America	236	Database of patients' samples referred for clinical diagnosis or research to Mayo Clinic Genomics Laboratory	1.27% (*n =* 3) 2 with more than 2,000 repeats, 1 with 80–100 repeats	0.42% (*n =* 1)	NA	NA	NA	Fragment length analysis on an 3730xl genetic analyzer. Then, analysis of repeat primed PCR. Expansions were identified and put forward for Southern with subsequent hybridization
Rikos et al. (2020)	Greece	74	HLD patients who underwent negative diagnostic genetic testing for HD at AHEPA University General Hospital and University Hospital of Larissa	1.35% (*n =* 1)	–	Choreiform movements of the extremities and depression Mother had cognitive decline at 55-60 years old	Male	48 years	First, PCR amplification and then, fragment length analysis on an ABIPrism 3730xl sequencer. Expansions were identified and put forward for Southern with subsequent hybridization
Kaur et al. (2020)	India	49	Unrelated cases referred (from major Indian neurological clinics/academic institutes) with clinical diagnosis of HD and its differentials enrolled in the CSIR-GOMED project	0%		NA	NA	NA	Repeat expansions in *C9orf72* were amplified by repeat-primed PCR. Then, PCR products were analyzed by capillary electrophoresis on the 3730xl genetic analyzer, and repeat sizes estimated using GeneMapper software

* *Summary statistics from patients harboring the C9orf72 hexanucleotide repeat expansion. HD, Huntington Disease; HLD, Huntington-Like Disorder; NA, Not applicable or not available; NHNN, Neurogenetics Unit of the National Hospital for Neurology and Neurosurgery, London*.

In two studies, published data were obtained from samples collected from HLD patients (Baine et al., 2018; Ida et al., 2018). In five studies, including the two previously mentioned articles, the diagnostic criteria for HLD were: (a) A clinical presentation consistent with Huntington disease when assessed by an experienced neurologist, neurogeneticist, or movement disorder specialist, and (b) A negative test for the expanded (CAG)n-*HTT* variant (<36 CAG repeats) or expanded (CTG)n-*JPH3* variants (Hensman Moss et al., 2014a; Kostic et al., 2014; Baine et al., 2018; Ida et al., 2018; Martins et al., 2018a). In three studies, the diagnostic criteria for HLD were the presence of a movement disorder consistent with Huntington disease phenotype and one or more of the following: cognitive impairment, behavioral or psychiatric symptoms, or a family history of psychiatric or neurological disorders compatible with dominant inheritance, and a negative test for the expanded (CAG)n-*HTT* variant (<36 CAG repeats) or expanded (CTG)n-*JPH3* variants, a causative mutation of HLD type 2 (Koutsis et al., 2015; Mariani et al., 2016; Rikos et al., 2020). Two studies did not mention the diagnostic criteria for HLD (Fedotova et al., 2016; Kaur et al., 2020). At least six studies described a diagnostic workup that excluded other genetic causes of HLD including neuroacanthocytosis, neurodegeneration with brain iron accumulation, *DJ-1* mutations, cerebral autosomal dominant arteriopathy with subcortical infarcts and leukoencephalopathy disease, HLD type 2, SCA-1, SCA-2, SCA-3, SCA-7, SCA-8, SCA-12, SCA-10, SCA-17, dentatorubral-pallidoluysian atrophy, and familial prion disease (Kostic et al., 2014; Koutsis et al., 2015; Mariani et al., 2016; Baine et al., 2018; Martins et al., 2018a; Rikos et al., 2020).

*C9orf72* genotyping assays in six studies were carried out by fragment analysis and repeat-primed PCR. Four out of 10 studies performed Southern blot for repeats sizing when a C9orf72 repeat expansion was detected (Hensman Moss et al., 2014b; Kostic et al., 2014; Ida et al., 2018; Rikos et al., 2020). Six studies reported at least one HLD patient carrying the *C9orf72* repeat expansion. Three studies reported at least one HLD patient carrying a *C9orf72* intermediate allele. In total, five affected HLD individuals harbor an intermediate allele.

The average age at onset of patients carrying the *C9orf72* hexanucleotide repeat expansion was 46.8 years. Only four studies reported the gender of affected individuals, where three out of five positive cases are women. Psychiatric symptoms, chorea-type motor symptoms, and parkinsonism symptoms predominated in affected individuals harboring the pathogenic repeat expansion.

### Study Quality

We evaluated eight cross-sectional and two case-control studies. Of the eight cross-sectional studies, six of them got seven out of 10 stars (good quality), whereas the remaining studies achieved six stars and four stars (low quality). Additionally, one case-control study received seven out of nine stars (good quality), and the other case-control study obtained six stars (good quality) (Table 2).

**Table 2:** Quality assessment of each article included in the systematic review.

**References**	**Study design**	**Selection**				**Comparability**	**Outcome**			**Total stars**
		**Representati-veness of the sample**	**Sample size justification**	**Exclusion of other relevant diseases**	**Ascertainment of exposure (*C9orf72*)**	**Control for confounding factors**	**Assessment of outcome (HLD diagnosis)**	**Statistical test**		
Hensman Moss et al. (2014a)	Cross-sectional	+			++		++	+		6/10
Kostic et al. (2014)	Cross-sectional	+		+	++		++	+		7/10
Koutsis et al. (2015)	Cross-sectional	+		+	++		++	+		7/10
Mariani et al. (2016)	Cross-sectional	+		+	++		++	+		7/10
Martins et al. (2018a)	Cross-sectional	+		+	++		++	+		7/10
Baine et al. (2018)	Cross-sectional	+		+	++		++	+		7/10
Ida et al. (2018)	Cross-sectional	+		+	++		++	+		7/10
Kaur et al. (2020)	Cross-sectional	+			++			+		4/10
**References**	**Study design**	**Selection**				**Comparability**	**Exposure**			**Total stars**
		**Case definition (HLD diagnosis)**	**Representativeness of the cases to target population**	**Selection of community-based controls**	**Definition of controls**	**Control for confounding factors**	**Ascertainment of exposure (*****C9orf72*****)**	**Same method used for cases and controls**	**All cases and controls were assessed for exposure**	
Rikos et al. (2020)	Case-control	+	+	+	+		+	+	+	7/9
Fedotova et al. (2016)	Case-control		+	+	+		+	+	+	6/9

*HLD, Huntington-Like Disorder*.

### Effect on Outcomes

**Figure 2:**
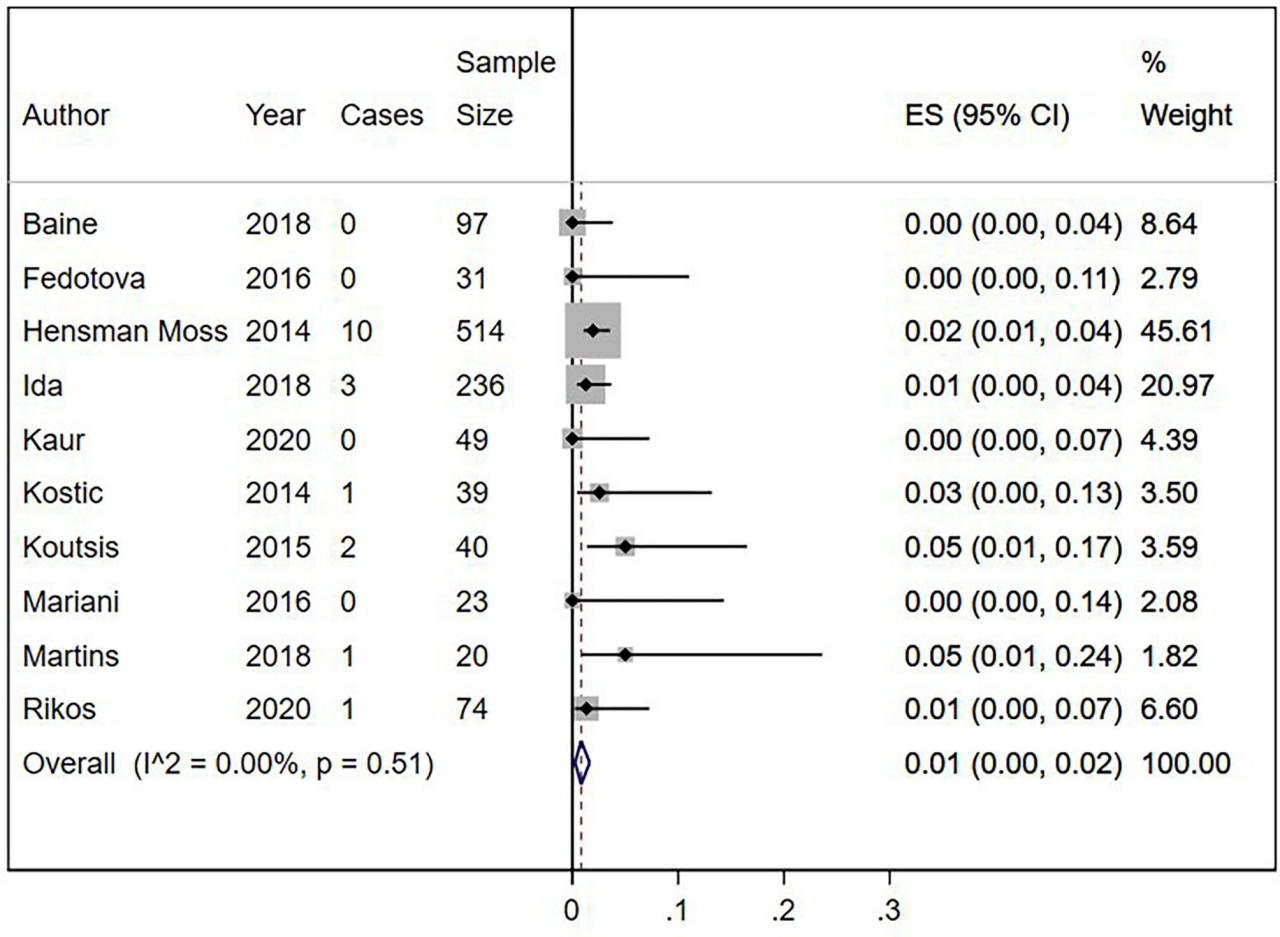
Percentage of *C9orf72* hexanucleotide repeat expansion in Huntington Disease-Like patients. Meta-analysis of 10 studies for prevalence of *C9orf72* hexanucleotide full repeat expansion in Huntington Disease-Like patients. ES, Estimated proportion; 95% CI, 95% Confidence interval.

**Figure 3:**
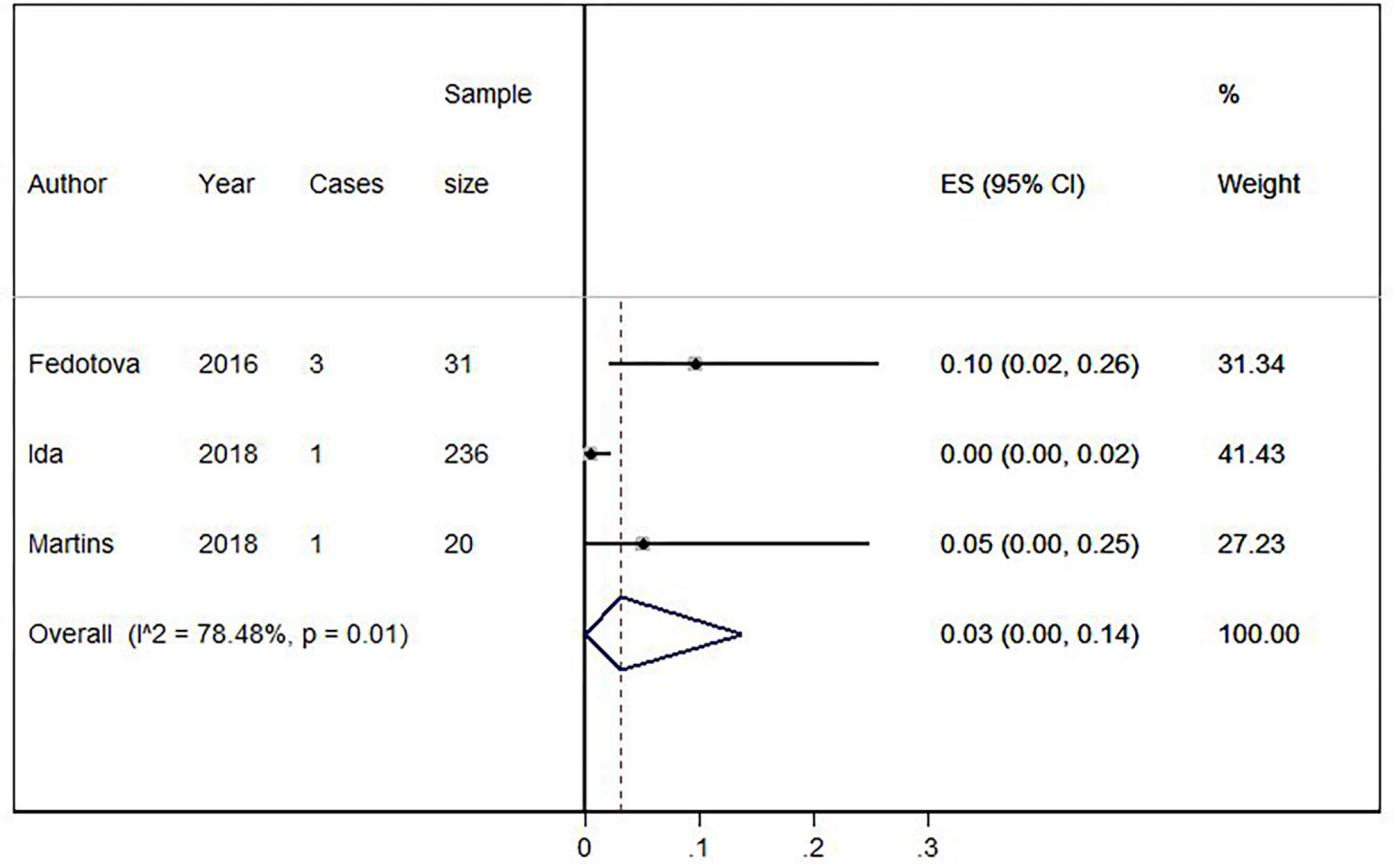
Percentage of *C9orf72* “intermediate allele” expansion in Huntington Disease-Like patients. Meta-analysis of three studies for prevalence of *C9orf72* hexanucleotide “intermediate allele” repeat expansion in Huntington Disease-Like patients. ES, Estimated proportion; 95% CI, 95% Confidence interval.

Eighteen out of 1,123 HLD patients had a *C9orf72* hexanucleotide repeat expansion. We conducted a cumulative analysis of the nine studies, including studies that reported no HLD patients with the *C9orf72* hexanucleotide repeat expansion. The overall frequency of the expansion was 1.0% (95% CI: 0–2%, *I*^2^ = 0%, *p* = 0.51) (Figure 2). Also, we conducted a cumulative analysis of three studies reporting HLD patients carrying “intermediate” alleles of C9*orf* 72 (Fedotova et al., 2016; Ida et al., 2018; Martins et al., 2018a). The overall frequency of the *C9orf72* intermediate allele was 3% (95% CI: 0–14%; *I*^2^ = 78.48%; *p* = 0.01) (Figure 3).

### Sensitivity Analysis

**Figure 4:**
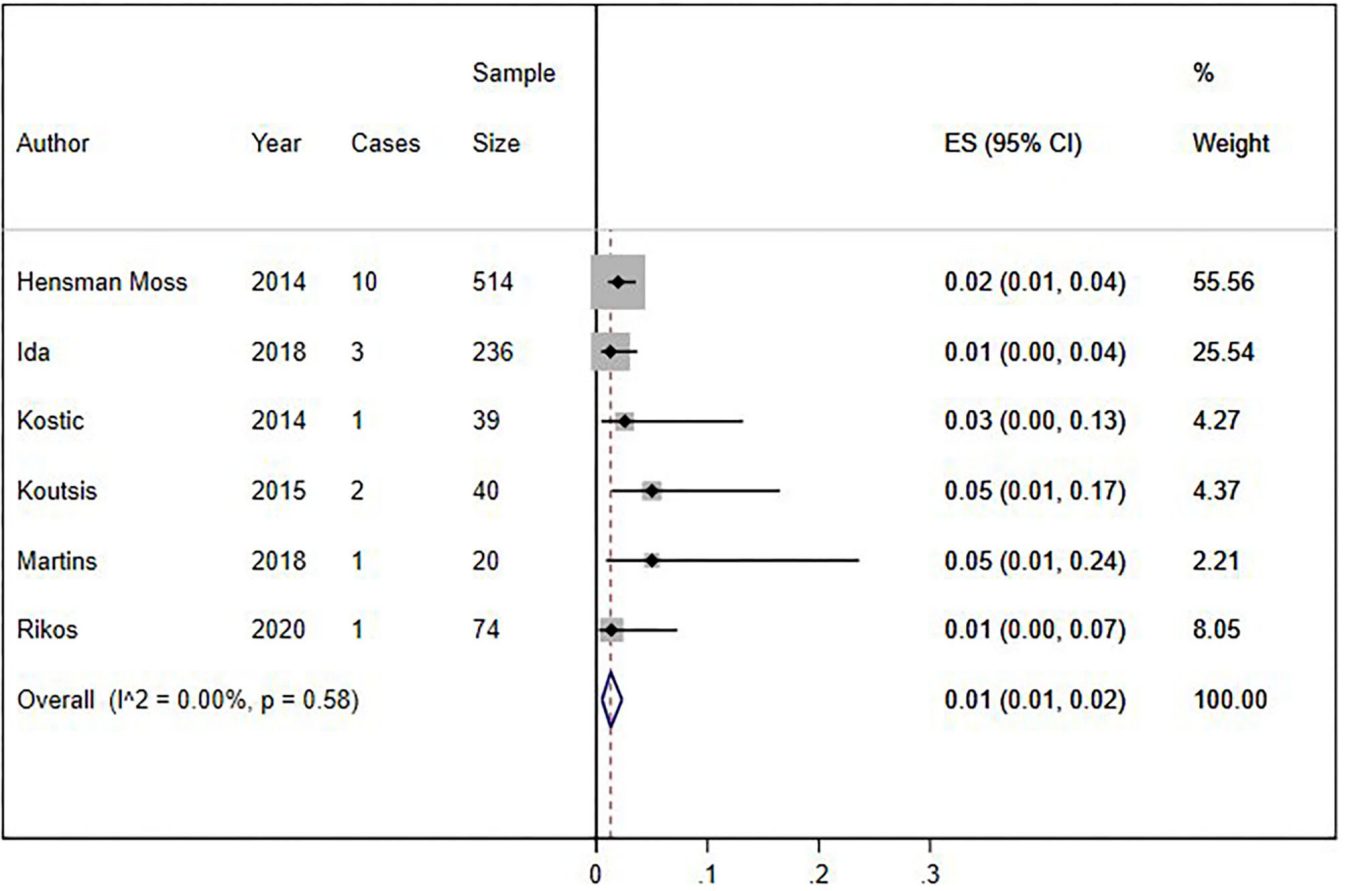
Sensitivity analysis of percentage of *C9orf72* hexanucleotide repeat expansion in Huntington Disease-Like patients, without studies with zero results. Meta-analysis of six studies for prevalence of *C9orf72* hexanucleotide repeat expansion in Huntington Disease-Like patients, excluding articles which report no *C9orf72* hexanucleotide repeat expansion patient. ES, Estimated proportion; 95% CI, 95% Confidence interval.

We conducted a cumulative analysis of six studies, excluding studies that reported no HLD patients with the *C9orf72* hexanucleotide repeat expansion (Fedotova et al., 2016; Mariani et al., 2016; Baine et al., 2018). The overall frequency of the expansion was 1% (95% CI: 1–2%, *I*^2^ = 0%, *p* = 0.58) (Figure 4). Additionally, we calculated the overall frequency of the *C9orf72* expansion by study type. Case-control studies (Fedotova et al., 2016; Rikos et al., 2020) showed 1% frequency (95% CI: 0–4%, *I*^2^ was not calculated), representing 9.40% of all weighted studies. Cross-sectional studies (Hensman Moss et al., 2014a; Kostic et al., 2014; Koutsis et al., 2015; Mariani et al., 2016; Baine et al., 2018; Ida et al., 2018; Martins et al., 2018a) also showed 1% (95% CI: 0–2%, *I*^2^ = 12.63%, *p* = 0.33), representing 90.6% of all weighted studies.

## Discussion

This systematic review was conducted in four primary publication databases and found 10 studies that reported the *C9orf72* pathogenic repeat expansion frequency among patients diagnosed with HLD (Hensman Moss et al., 2014a; Kostic et al., 2014; Koutsis et al., 2015; Fedotova et al., 2016; Mariani et al., 2016; Baine et al., 2018; Ida et al., 2018; Martins et al., 2018a; Rikos et al., 2020). Most of these cases were referred with a clinical diagnosis of Huntington Disease by an expert neurologist or neurogeneticist for the (CAG)n-*HTT* expansion test. Most of these studies were conducted in European countries (Hensman Moss et al., 2014a; Kostic et al., 2014; Koutsis et al., 2015; Fedotova et al., 2016; Mariani et al., 2016; Martins et al., 2018a; Rikos et al., 2020). The estimated frequency of the expansion is around 1% among populations from Europe, South Africa, India, and the United States. An additional 3% of HLD cases (5/287) carry intermediate-sized alleles in the *C9orf72* gene (20–29 repeats). There have been two recent reviews published on the phenotypic expression of C9orf72 (Marogianni et al., 2019; Rikos et al., 2020). However, we highlight that we have strictly followed the PRISMA guidelines for systematic reviews and performed a separate analysis for intermediate allele cases.

The frequency of *C9orf72* expansion among patients diagnosed with HLD is understudied in many regions across the globe. For example, most Asian nations were not represented in this review. Other regions like Latin America and the Caribbean with disparities in genetic testing availability do not report HLD cases harboring this repeat expansion (Walker et al., 2018). Despite this, our systematic review suggests that there may be differences in the frequency of the *C9orf72* repeat expansion as a cause of HLD among different ethnic groups.

Most positive cases were reported to harbor complex phenotypes combining motor phenomena with an unusually high frequency of psychiatric disorders. The classical chorea presentation was not common among these cases. Instead, these patients manifested abnormal movement disorders described as myoclonus, dystonia, and tremor or even no movement disorder at all. There are no established criteria for when genetic testing of Huntington Disease should be performed. The studies included in our analysis did not define their threshold for performing genetic testing of a patient. Thus, since *C9orf72* pathogenic repeat expansion is present in patients with frontotemporal dementia, amyotrophic lateral sclerosis, and atypical parkinsonism (Prado et al., 2015; Wilke et al., 2016; Zou et al., 2017), it remains unclear whether Huntington-like features should be considered as a separate phenotypic manifestation of *C9orf72* repeat expansion or as an atypical expression of neurodegenerative diseases among *C9orf72* cases.

The interpretation of reported HLD cases with the co-existence of an “intermediate allele” range is challenging. Of the two out of three publications that found cases carrying a 27 hexanucleotide repeat expansion (Fedotova et al., 2016; Martins et al., 2018a), only one of these cases was associated with perioral choreic movements, memory loss, executive dysfunction, and psychiatric symptomatology (Martins et al., 2018a). It is not clear whether buccolingual chorea was related or not to previous “transient” treatment with dopamine D2 receptor blockers. Some authors have demonstrated that hexanucleotide repeats in the low range are stable across generations and present in non-affected control populations (Ng and Tan, 2017; Van Mossevelde et al., 2017). However, others suggest a slightly increased risk of developing complex disorders with a high frequency of psychiatric disturbances (Ng and Tan, 2017; Martins et al., 2018a). This association was not assessed because of the null report of clinical findings in the included studies. Other genetic and epigenetic factors like methylation of larger expansion, incomplete penetrance, and somatic/germline mosaicism (Ng and Tan, 2017) further complicate accurate estimation of pathogenicity of “intermediate alleles.”

We evaluated the quality of evidence and precision of the estimates using the Newcastle Ottawa instrument (Stang, 2010; Modesti et al., 2016). Based on this scale, eight of the studies selected to estimate the frequency of *C9orf72* hexanucleotide repeat expansion in HLD patients were designated as showing good performance or quality. Furthermore, all the studies reporting on *C9orf72* intermediate alleles were found to have good performance. Five out of seven cross-sectional studies assessed for other relevant causes for HLD were excluded (Kostic et al., 2014; Koutsis et al., 2015; Mariani et al., 2016; Baine et al., 2018; Martins et al., 2018a). All the selected studies lacked representativeness to a target population (external validity), but all included the total available population into the analysis, keeping the sample's overall representativeness. However, none of these studies justified their sample size. Finally, because most studies aim to determine the prevalence of *C9orf72* hexanucleotide repeat expansion in HLD patients, none of these controlled for confounding factors. The overall confidence in the estimation of the results was adequate.

Concerning the accuracy of the calculated results, we found that seven of the nine studies had a small sample size (<100 patients), which could contribute to the imprecision in estimating the effect. The heterogeneity was low (<40%) when including all nine studies, and when excluding studies that did not report HLD patients with a *C9orf72* hexanucleotide repeat expansion. Otherwise, heterogeneity for the analysis with intermediate alleles was considerable (>75%), perhaps because of their ethnic background differences. This meta-analysis included three studies, and each of them included a different country population: Portugal (Martins et al., 2018a), United States (Ida et al., 2018), and Russia (Fedotova et al., 2016). The low heterogeneity allows us to have greater confidence in estimating the effect. So, the frequency of the *C9orf72* hexanucleotide repeat expansion within the HLD population could be close to the actual frequency of this mutation in patients with HLD, despite some representativeness limitations of all studies.

Our systematic review's main limitations were the small number of published studies, the lack of uniform criteria for the clinical diagnosis of HLD, and their concentration within developed countries. The relatively small sample sizes further complicated these estimates, although it is worth noting that subjects with HLD are considered rare. There are no published cases reporting choreic movements among *C9orf72* amyotrophic lateral sclerosis/frontotemporal dementia cases (Yokoyama et al., 2014; Bourinaris and Houlden, 2018). However, relaxed clinical criteria for HLD might include other related neurodegenerative disorders, including the frontotemporal dementia-motor neuron disease spectrum, so these diagnoses cannot be ruled out in the included patients. Although only four studies used the gold-standard Southern blot for counting the repeat expansions, all studies used a repeat-primed PCR, which had an overall good performance in diagnosis (Cleary et al., 2016). There still exists, however, the chance of false positive or false negative results (Akimoto et al., 2014). Additionally, some regions like Africa, Asia, and Latin-America are not represented in this review. Furthermore, sources of heterogeneity in these studies attributed to differences in populations and study designs (cross-sectional and case-control) might negatively affect our estimates' accuracy, limiting their generalization.

## Conclusions and Research Recommendations

A small number of studies report the frequency rate of *C9orf72* hexanucleotide repeat expansion among HLD patients. These are mostly of good quality, but with a low external validation. Nevertheless, *C9orf72* repeat expansions do account for a small percentage of such cases. Intermediate allele expansions in this gene may also be related to HLD cases. Additionally, the frequency of *C9orf72* expansion among HLD cases should be further investigated in African, Asian, and Latin American populations to confirm this observation.

## Data Availability Statement

Publicly available datasets were analyzed in this study. This data can be found at: https://figshare.com/articles/dataset/C9orf72_hexanucleotide_repeat_in_Huntington-like_patients_Systematic_review_and_meta-analysis/12057759.

## Author Contributions

CA-D, AR-V, and MC-O conceptualized the idea. JP-M designed the search strategy, made, and organized the systematic research. CA-D, CA-R, and KP-B reviewed the citations and extracted the data. CA-R and CA-D made the statistical analysis. CA-D, CA-R, KP-B, NM, AR-V, and MC-O wrote the first draft. All the authors interpreted the results, contributed to manuscript revision, participated in the manuscript redaction, read, and approved the submitted version.

## Conflict of Interest

BT holds patents on the clinical testing and therapeutic intervention for the hexanucleotide repeat expansion of C9orf72. MC-O is a review editor in Frontiers: Neurology Neurogenetics. The remaining authors declare that the research was conducted in the absence of any commercial or financial relationships that could be construed as a potential conflict of interest.

## Abbreviations

HLD: Huntington-like disorders
SCA: Spinocerebellar ataxia.
