# Supplementary material for: C9orf72 Hexanucleotide Repeat in Huntington-Like Patients: Systematic Review and Meta-Analysis
Source: Front Genet. 2020 Nov 2;11:551780. doi: 10.3389/fgene.2020.551780 (PMC7667021; doi:10.3389/fgene.2020.551780)
Supplement: Supplementary file 1 [file Table_1.DOCX]

**Supplementary Material 1: Research strategies**

**Web of Science (n=57)**

| #1 | TS=(“Huntington Disease”) OR TS=(Huntington* NEAR/3 Disease) OR TS=(Huntington* NEAR/3 Chorea) OR TS=(“Huntington Chorea”) OR TS=(“Huntington's Disease”) OR TS=(Huntington's Chorea) OR TS=(“Chronic Progressive Hereditary Chorea”) OR TS=(“Chronic Progressive” NEAR/3 Chorea) |
| --- | --- |
| #2 | TS=(“Huntington Disease-Like”) OR TS=(“Huntington Disease Phenocopy”) OR TS=(“Huntington's Disease Phenocopies”) OR TS=(“Huntington-Like”) OR TS=(“Prion Disease” AND “Early-Onset” AND “Prominent Psychiatric Features”) OR TS=(huntingtin) |
| #3 | TS=("C9orf72 protein") OR TS=("chromosome 9 open reading frame 72 protein") OR TS=(C9orf72) OR TS=("chromosome 9") OR TS=("9p-linked") OR TS= ( chromosome NEAR/2 9) |
| #4 | (#1 OR #2 ) AND #3 |

**Medline via Web of Science (n=36)**

| #1 | TS=(“Huntington Disease”) OR TS=(Huntington* NEAR/3 Disease) OR TS=(Huntington* NEAR/3 Chorea) OR TS=(“Huntington Chorea”) OR TS=(“Huntington's Disease”) OR TS=(Huntington's Chorea) OR TS=(“Chronic Progressive Hereditary Chorea”) OR TS=(“Chronic Progressive” NEAR/3 Chorea) |
| --- | --- |
| #2 | TS=(“Huntington Disease-Like”) OR TS=(“Huntington Disease Phenocopy”) OR TS=(“Huntington's Disease Phenocopies”) OR TS=(“Huntington-Like”) OR TS=(“Prion Disease” AND “Early-Onset” AND “Prominent Psychiatric Features”) OR MH=(Huntington Disease ) OR TS=(huntingtin) |
| #3 | TS=("C9orf72 protein") OR TS=("chromosome 9 open reading frame 72 protein") OR TS=(C9orf72) OR TS=("chromosome 9") OR TS=("9p-linked") OR TS= ( chromosome NEAR/2 9) OR MH=(C9orf72 Protein ) |
| #4 | (#1 OR #2 ) AND #3 |

**Embase (n=35)**

| #1 | 'huntington chorea'/exp OR 'huntington chorea' OR 'huntington disease like syndrome'/exp OR 'huntington disease like syndrome' OR 'huntingtin'/exp OR huntingtin |
| --- | --- |
| #2 | 'c9orf72 gene'/exp OR 'c9orf72 gene' |
| #3 | #1 AND #2 |

**SCOPUS (n=82)**

( ( TITLE-ABS-KEY ( "Huntington Disease-Like" ) OR TITLE-ABS-KEY ( "Huntington Disease Phenocopy" ) OR TITLE-ABS-KEY ( "Huntington's Disease Phenocopies" ) OR TITLE-ABS-KEY ( "Huntington-Like" ) OR TITLE-ABS-KEY ( "Prion Disease" AND "Early-Onset" AND "Prominent Psychiatric Features" ) OR TITLE-ABS-KEY ( huntingtin ) ) OR ( TITLE-ABS-KEY ( "Huntington Disease" ) OR TITLE-ABS-KEY ( huntington* PRE/3 disease ) OR TITLE-ABS-KEY ( huntington* W/3 disease ) OR TITLE-ABS-KEY ( huntington* PRE/3 chorea ) OR TITLE-ABS-KEY ( huntington* W/3 chorea ) OR TITLE-ABS-KEY ( "Huntington Chorea" ) OR TITLE-ABS-KEY ( "Huntington's Disease" ) OR TITLE-ABS-KEY ( "Huntington's Chorea" ) OR TITLE-ABS-KEY ( "Chronic Progressive Hereditary Chorea" ) OR TITLE-ABS-KEY ( "Chronic Progressive" PRE/3 chorea ) OR TITLE-ABS-KEY ( "Chronic Progressive" W/3 chorea ) ) ) AND ( TITLE-ABS-KEY ( "C9orf72 protein" ) OR TITLE-ABS-KEY ( "chromosome 9 open reading frame 72 protein" ) OR TITLE-ABS-KEY ( c9orf72 ) OR TITLE-ABS-KEY ( "chromosome 9" ) OR TITLE-ABS-KEY ( "9p-linked" ) OR TITLE-ABS-KEY ( chromosome PRE/2 9* ) OR TITLE-ABS-KEY ( chromosome W/2 9* ) )
