# Supplementary material for: C9orf72 Hexanucleotide Repeat in Huntington-Like Patients: Systematic Review and Meta-Analysis
Source: Front Genet. 2020 Nov 2;11:551780. doi: 10.3389/fgene.2020.551780 (PMC7667021; doi:10.3389/fgene.2020.551780)
Supplement: Supplementary file 2 [file Table_2.DOCX]

**Supplementary Material 2**

**Table S1: Descriptive information on each excluded article in the systematic review.**

| **Author and year (Ref)** | **Country** | **Reason for exclusion** |
| --- | --- | --- |
| Hensman Moss 2014 (26) | United Kingdom | Conference abstract presenting results that already are published in other document included in the systematic review |
| Abramycheva 2015 (24) | Russia | Conference abstract presenting results that already are published in other document included in the systematic review |
| Dolzhenko 2017 (25) | Netherlands | Not included Huntington-Like patients in the study |
| Kartanou 2017 (27) | Greece | It is a scoping review |
| Martins 2017 (28) | Portugal | Conference abstract presenting results that already are published in other document included in the systematic review |
| Marogianni 2019 (14) | Greece | It is a systematic review with no new data presented |
| Beck 2013 (30) | United Kingdom | Original research that analyzed the same database from Hensman Moss’ study (10). Beck study was excluded because it included a smaller sample size. |
